# Acceptance of a Smartphone-Based Visual Field Screening Platform for Glaucoma: Pre-Post Study

**DOI:** 10.2196/26602

**Published:** 2021-09-17

**Authors:** Esmael Kedir Nida, Sisay Bekele, Luc Geurts, Vero Vanden Abeele

**Affiliations:** 1 e-Media Lab KU Leuven Leuven Belgium; 2 Department of Ophthalmology Institute of Health Jimma University Jimma Ethiopia

**Keywords:** mHealth acceptance, UTAUT, glaucoma screening, mhealth for eye care, mhealth, glaucoma, visual, eye, ophthalmology, ophthalmic, mobile phone

## Abstract

**Background:**

Glaucoma, *the silent thief of sight*, is a major cause of blindness worldwide. It is a burden for people in low-income countries, specifically countries where glaucoma-induced blindness accounts for 15% of the total incidence of blindness. More than half the people living with glaucoma in low-income countries are unaware of the disease until it progresses to an advanced stage, resulting in permanent visual impairment.

**Objective:**

This study aims to evaluate the acceptability of the Glaucoma Easy Screener (GES), a low-cost and portable visual field screening platform comprising a smartphone, a stereoscopic virtual reality headset, and a gaming joystick.

**Methods:**

A mixed methods study that included 24 eye care professionals from 4 hospitals in Southwest Ethiopia was conducted to evaluate the acceptability of GES. A pre-post design was used to collect perspectives before and after using the GES by using questionnaires and semistructured interviews. A Wilcoxon signed-rank test was used to determine the significance of any change in the scores of the questionnaire items (two-tailed, 95% CI; *α*=.05). The questionnaire and interview questions were guided by the Unified Theory of Acceptance and Use of Technology.

**Results:**

Positive results were obtained both before and after use, suggesting the acceptance of mobile health solutions for conducting glaucoma screening by using a low-cost headset with a smartphone and a game controller. There was a significant increase (two-tailed, 95% CI; *α*=.05) in the average scores of 86% (19/22) of postuse questionnaire items compared with those of preuse questionnaire items. Ophthalmic professionals perceived GES as easy to use and as a tool that enabled the conduct of glaucoma screening tests, especially during outreach to rural areas. However, positive evaluations are contingent on the accuracy of the tool. Moreover, ophthalmologists voiced the need to limit the tool to screening only (ie, not for making diagnoses).

**Conclusions:**

This study supports the feasibility of using a mobile device in combination with a low-cost virtual reality headset and classic controller for glaucoma screening in rural areas. GES has the potential to reduce the burden of irreversible blindness caused by glaucoma. However, further assessment of its sensitivity and specificity is required.

## Introduction

### Background

Glaucoma is the leading cause of irreversible blindness worldwide, affecting approximately 64 million people [1,2]. A large proportion of glaucoma cases worldwide are undiagnosed or suboptimally managed [3]. More than half of the people living with the disease in low-income countries are unaware of the condition until it progresses to an advanced stage resulting in visual impairment [4,5]. As blindness caused by glaucoma is irreversible, early detection of the disease is critical [6]. Visual field testing (VFT) is one of the major tests used for the screening and diagnosis of glaucoma [7]. The test assesses central and peripheral vision of each eye separately to detect vision loss, which, in case of glaucoma, gradually progresses from the periphery to the center. The test is mostly performed using standard automated perimetry (SAP) equipment, which is expensive and not easily portable [8]. During the test, the patient is expected to look at the center of a dimly lit bowl-shaped area and press a response button upon seeing a small oval light appearing briefly at different places in the field of view [9]. The equipment records the seen and unseen lights and at the end of the test, provides a result representing the visual field status of each eye. For people living in rural areas in low-income countries with limited access to ophthalmic care, glaucoma screening and diagnosis testing through VFT is nearly nonexistent [5]. If an affordable alternative to the SAP equipment were to become available, it is likely that more glaucoma cases can be detected, especially in rural areas where the burden of the disease is most significant [10].

Mobile health (mHealth)—the use of mobile computing and communication technologies in health care and public health—is an emerging field. Its potential for improving health care delivery has been well demonstrated [11-13]. mHealth interventions have proven particularly successful in improving access to eye care in low-income countries challenged by a lack of eye care professionals [14]. Recent efforts have focused on designing affordable and portable technology for conducting VFT using tablets [15] or smartphones with virtual reality (VR) headsets [16]. Thus far, studies evaluating these VFT solutions are limited to clinical validation of prototypes in high-income countries, comparing their results with those of the gold standard SAP equipment. SAP equipment costs around US $20,000, whereas the cost of a stereoscopic headset, smartphone, and gaming joystick is approximately US $350 at current prices; hence, only a fraction of the cost of the gold standard. However, such low-cost and portable alternatives are to be used in rural areas of ow-income countries. The context of these areas is unique in terms of technology adoption, presenting challenges such as technological ineptitudes or obstructive cultural beliefs [17,18]. The successful adoption of new technology for health care in low-income countries is highly dependent not only on clinical accuracy but also on, for example, perceived ease of use and alignment with available infrastructure and protocols [19,20]. Hence, complementary to verifying the clinical accuracy of new technological innovations for health care, an assessment of the drivers for user acceptance is crucial.

**Figure 1:**
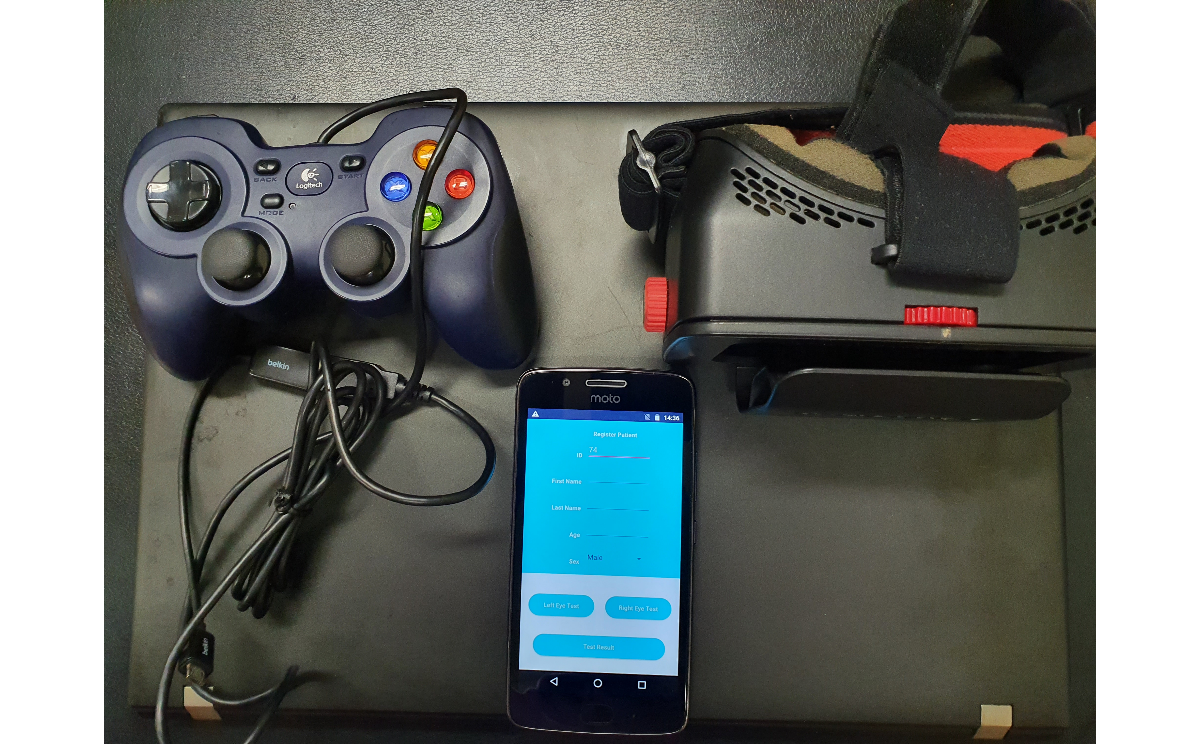
The three components of Glaucoma Easy Screener: smartphone app, virtual reality headset, and joystick.

We conducted a mixed methods study to evaluate the acceptability of the Glaucoma Easy Screener (GES), a low-cost and portable visual field screening platform comprising a smartphone, a stereoscopic headset, and a gaming joystick (Figure 1). The study included 24 eye care professionals from 4 hospitals in Southwest Ethiopia. A pre-post design was employed to collect their perspectives before and after using the GES, using questionnaires and semistructured interviews. The questionnaire and interview questions were guided by the Unified Theory of Acceptance and Use of Technology (UTAUT) [21], which is widely applied to study the adoption of technology. Previous studies have demonstrated the applicability of the model for the adoption of mHealth and eHealth in low-income countries [17,22,23]. The specific research aims of this study were (1) to assess the acceptability of GES and (2) to identify potential challenges that might affect the adoption of GES. Findings from this study suggest acceptance of GES for glaucoma screening, even in rural areas of low-income countries where patients lack technological aptitude. Nevertheless, our findings also highlight the importance of using the tool for screening only and not for diagnosis, and the importance of achieving adequate accuracy.

### Related Work

A number of studies exist on the use and acceptance of mHealth in the context of low-income countries. mHealth solutions have been proposed for several health care interventions, such as support for patients with HIV [23], neonatal child care [24], and diagnosis and treatment of pneunomia [25]. Although most of these studies show positive results for the acceptability and usability of mHealth in low-resource settings such as sub-Saharan Africa, their findings cannot be generalized for eye care.

To the best of the authors’ knowledge, there are only 2 studies on the acceptability and usability of mHealth for eye care in middle-or limited-income countries. Lodhia et al [24] investigated the acceptability and usability of the portable eye examination kit, a smartphone-based comprehensive ophthalmic examination system with clip-on hardware to examine eye diseases such as cataract, which was deployed in Kenya. On the basis of qualitative analysis of interviews with patients, health care providers, and key decision makers in ophthalmic care, the study found that using portable eye examination kit patients were able to overcome the barriers to accessing ophthalmic services. The acceptability of the solution to health care professionals was further demonstrated by their perceived ability to use the solution easily. However, deployment challenges identified by the study, such as the need for governmental support, further training for health care professionals, ensuring data protection, and access to smartphones at low cost. Ludwig et al [25] evaluated the feasibility of a smartphone-based ophthalmic imaging system (eyeGo) in India. The study found that ophthalmic professionals (OPs) learned to use the system quickly. Patients also found it comfortable during the imaging process. The above 2 studies show encouraging results for the feasibility and potential acceptability of mHealth-based solutions for eye care in low-income countries. However, both studies addressed the application of smartphones for ophthalmic imaging to examine diseases such as cataracts. More evidence is required on the acceptability and usability of mHealth to perform visual field screening tests, which play a key role in the screening and diagnosis of other eye diseases such as glaucoma.

### The UTAUT

**Figure 2:**
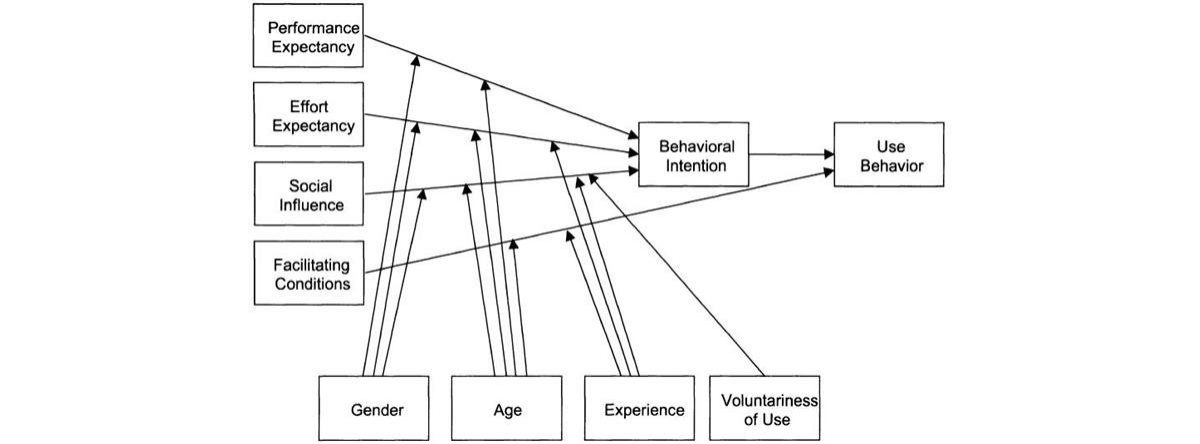
The Unified Theory of Acceptance and Use of Technology model by Venkatesh et al [21].

The UTAUT is widely applied to study the adoption of technology [21] and is also used in a health context [26-32]. The UTAUT model identifies four drivers of technology adoption. Performance expectancy (PE) is the degree to which a user or potential user of an information system believes that the system will contribute to the attainment of some benefits related to his or her job performance. Effort expectancy (EE) is the degree to which an information system is easy to use. Social influence (SI) refers to the degree to which individuals perceive that influential people believe they should use a new information system. Facilitating conditions (FCs) are defined as the extent to which an individual believes that there is technical and organizational infrastructure to support the use of an information system. These four drivers are direct determinants of behavioral intention (BI) and, ultimately, use-behavior (Figure 2). In addition, gender, age, experience, and voluntariness of use were posited to moderate the four constructs.

Gender, age, experience, and voluntariness of use were posited to moderate the four constructs.

Several studies have applied this model to scrutinize the adoption of mHealth and eHealth in various contexts, including in low-income countries [17,22,23,33]. Most of these studies adapted and extended the UTAUT model by tailoring items or adding items from the original study [21] to cater to specific technology categories or application domains [34,35].

To the best of our knowledge, no studies have investigated the acceptance of mHealth among eye health care professionals in the context of glaucoma screening. Given the unique context of designing mHealth solutions for glaucoma-related eye care in low-income countries, further evidence is required from different countries to guide the design of mHealth for eye care in various contexts.

## Methods

### Overview

In this study, the acceptance of a smartphone-based visual field-testing platform for glaucoma screening was evaluated through a combination of a UTAUT-based questionnaire and semistructured interviews. A pre-post design was applied to assess the changes in attitudes toward GES after practical use. We assessed the initial attitude toward the glaucoma screener of 24 OPs who had never used an mHealth glaucoma screener. Furthermore, we assessed changes in attitudes after the first use of the screener. A qualitative analysis of the interviews was used to further elaborate the quantitative data and gain additional feedback. This study was approved by the ethical review board of the Institute of Health, Jimma University. Written informed consent was obtained from all participants.

### The GES

The GES combines a smartphone app that runs on the Android platform, an ordinary gaming joystick, and a VR headset to perform quick visual field screening tests (Figure 1). The app was designed on the basis of user and task analysis of eye care in Southwest Ethiopia [36], to be used as a first-hand screening tool for diseases such as glaucoma. Given the limited access to primary eye care in these areas because of a shortage of both OPs and diagnostic equipment [36], GES enables local health centers to screen for potential cases and make more informed referral decisions.

**Figure 3:**
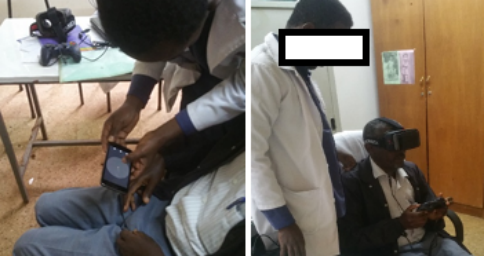
Left: An ophthalmic nurse demonstrating the test procedure to a patient using the demo app. Right: The ophthalmic nurse is monitoring the progress of the test with Glaucoma Easy Screener.

As VFT is a subjective test performed by the patient, a clear understanding of the test procedures is required to obtain reliable results. Patients are expected to stay focused and respond to the stimuli presented, and the test mainly relies on the performance of the patient. The role of the OP is to monitor the progress of the test and guide the patient to perform the test. Patients performing VFT for the first time are given an explanation about the test, mostly focusing on the need for fixation of the eye and providing responses only after perceiving a stimulus. GES includes a demonstration animation to help OPs explain the test procedure to patients. The demo animates the actual test environment with stimuli flashing at different locations on the screen around a central fixation target. During the test, patients wore the VR headset and responded to perceived stimuli using a joystick (Figure 3).

**Figure 4:**
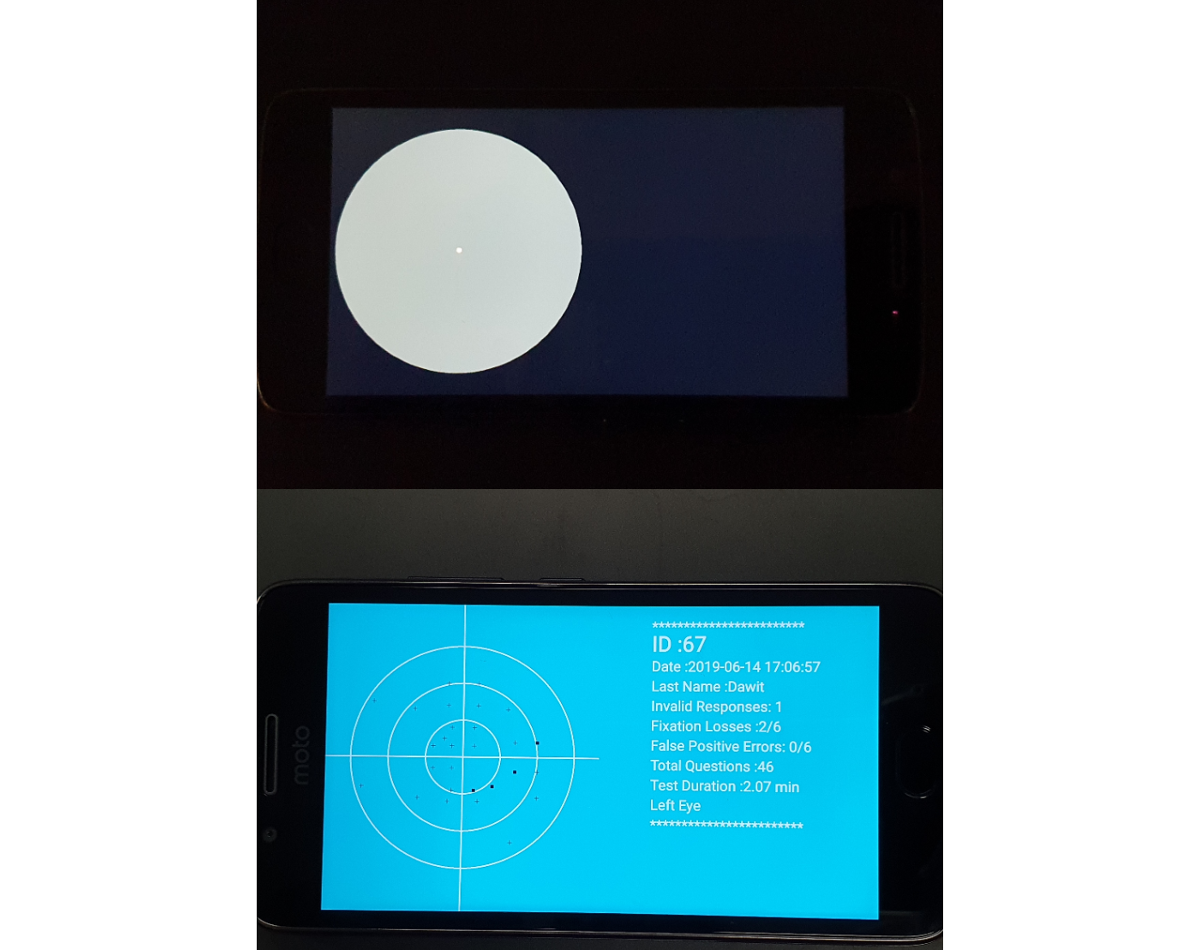
The test scene for the left eye is shown at the top with a central fixation target, and a sample result from the Glaucoma Easy Screener test is shown on the bottom. For the test results, plus ("+") signs represent seen (normal) locations and black squares represent missed (abnormal) locations.

Each eye is tested separately. A sound marks the end of the test for each eye, upon which the OP is required to take off the headset and start the test for the other eye. The test duration per eye ranges from 2 minutes (for those with no visual field defect) to 5 minutes (for those with an advanced visual field defect). At the end of the full test, the OPs check the test results for each eye. The screening results are presented on the smartphone screen by plotting seen and missed stimuli along with other test parameters, such as test duration and reliability indicators (Figure 4).

### Participants and Setting

The study was conducted at 4 different hospitals in Southwest Ethiopia in August and September 2018. The first hospital was Jimma University Medical Center in the town of Jimma, which is the only tertiary hospital in the southwest region. Jimma University Medical Center has an ophthalmology department providing tertiary eye care for a population of more than 20 million people in the region. Approximately 23 OPs who are usually involved in the screening and diagnosis of patients, including ophthalmic residents, ophthalmic nurses, optometrists, cataract surgeons, and opticians, were invited to participate in the study. Of these 23, 19 accepted the invitation. Each OP was asked to recruit a patient for the user test from among those who were screened that day. There were no specific inclusion criteria for the selection of patients, but preference was given to older patients from rural areas as they are expected to be the most challenging to supervise during VFT because of limited familiarity with technology. The study also included 3 other primary hospitals in the region: Saja Primary Hospital, Seka Chokorsa Primary Hospital, and Agaro Primary Hospital. Unlike Jimma University Medical Center, these primary hospitals do not have well-established eye units. They are also understaffed because of a shortage of OPs. All OPs working in these 3 primary hospitals accepted the invitation to participate in the study. There were only 2 ophthalmic nurses at Agaro Primary Hospital, and only one ophthalmic nurse at Saja Primary Hospital. Seka Chokorsa Primary Hospital relies on 2 integrated eye care worker (IECW) nurses with short ophthalmic training. They provide treatment for minor eye problems, such as trachoma, in addition to their regular duties. A procedure similar to that at the Jimma University Medical Center was followed for the participants at these 3 primary hospitals.

A total of 24 OPs completed both the preuse and postuse questionnaires and participated in the interviews (Table 1). Three additional OPs filled the preuse questionnaire but were not available to use GES; hence, they were excluded from the study.

**Table 1:** Participants’ profiles.

Characteristics			Frequency (N=24), n (%)
**Sex**			
	Male	22 (92)	
	Female	2 (8)	
**Age (years)**			
	20-30	17 (71)	
	31-40	6 (25)	
	61-70	1 (4)	
**Profession**			
	Ophthalmology resident	11 (46)	
	Optometrist	6 (25)	
	Ophthalmic nurse	4 (17)	
	IECW^a^ nurse	2 (8)	
	Cataract surgeon	1 (4)	

^a^IECW: integrated eye care worker.

### Procedure and Instruments

Participants completed a preuse questionnaire before the actual use of the GES. The aim of the preuse questionnaire was to evaluate OPs’ initial attitudes toward using a smartphone app for VFT, irrespective of the specific design and use experience with the prototype. The preuse questionnaire included items adapted to measure the UTAUT constructs in the context of glaucoma. The UTAUT items were taken from Venkatesh et al [21] but adapted to reflect the use of GES in the Ethiopian eye health care context (Table 2).

PE items assessed the perspective of OPs regarding the GES ability to perform reliable glaucoma screening. EE items assessed how easily OPs learned to use GES, how easily they administered the test for the patients, and their expectations regarding patients performing the test with minimal explanation. SI items assessed OP expectations regarding how other senior staff members and patients would perceive them because of the use of GES. FCs items assessed OP perspectives regarding the suitability of GES with the current infrastructure, environmental conditions, and availability of resources required for the successful use of GES in their workplace.

We also included items to assess the OPs’ overall BI to use GES. We added these items to cross-check the consistency of OP responses to the four constructs and their ultimate intention to adopt GES in their daily practice. A positive score for the four constructs and a negative response for BI or vice versa might indicate that additional factors need to be measured to explain acceptance of the technology.

**Table 2:** Mean scale scores for preuse and postuse.

Scale			Preuse score, mean (SD)		Postuse score, mean (SD)		Difference (95% CI)		*P* value^a^
**PE^b^**									
	PE1: This platform will be useful during outreach screening.	4.04 (0.81)		4.63 (0.49)		+0.58 (0.21 to 0.96)		.006	
	PE2: The platform will enable me to make more accurate diagnoses.	3.42 (0.72)		3.88 (0.95)		+0.46 (0.15 to 0.76)		.008	
	PE3^c^: I do not expect that this platform can be used for a reliable eye exam.	3.29 (0.95)		3.33 (0.96)		+0.04 (−0.54 to 0.62)		.89	
	PE4: More glaucoma cases can be detected by the platform.	3.38 (0.71)		4.17 (0.96)		+0.79 (0.34 to 1.24)		.005	
	PE5^d^: The results presented are informative enough to make referral decisions.	—^e^		4.21 (0.72)		—		—	
**EE^f^**									
	EE1: I think the platform will make it easy for me to administer visual field tests.	3.88 (0.61)		4.79 (0.41)		+0.92 (0.59 to 1.24)		<.001	
	EE2: I think learning how to use the platform will be easy.	4.00 (0.72)		4.71 (0.46)		+0.71 (0.44 to 0.97)		<.001	
	EE3: It will be easy to have patients wear the virtual reality headset.	3.63 (0.92)		4.42 (0.88)		+0.79 (0.42 to 1.16)		.001	
	EE4: Compared with the FDT^g^, administering visual field testing with this platform will be easier.	3.33 (0.82)		4.29 (0.85)		+0.96 (0.46 to 1.46)		.002	
	EE5: The platform will be easier for patients from rural areas compared with the FDT.	—		4.25 (1.19)		+0.58 (−0.01 to 1.18)		.047	
	EE6: Patients can easily use a joystick for responses.	3.71 (0.95)		4.25 (0.74)		+0.54 (0.15 to 0.94)		.01	
	EE7^d^: My interaction with the platform was clear and understandable.	—		4.42 (0.58)		—		—	
**SI^h^**									
	SI1: I think the senior ophthalmologist staff will recommend the platform for glaucoma screening.	3.67 (0.87)		4.08 (0.93)		+0.42 (−0.10 to 0.93)		.08	
	SI2: In general, I think the platform will be seen as useful by the ophthalmology department.	3.83 (0.76)		4.63 (0.49)		+0.79 (0.46 to 1.12)		.001	
	SI3: My colleagues will support my use of this platform.	3.67 (0.96)		4.46 (0.66)		+0.79 (0.38 to 1.20)		.002	
	SI4: My patients will be open to the use of this platform.	3.46 (0.98)		4.50 (0.59)		+1.04 (0.58 to 1.50)		.001	
	SI5: If I use the platform, I will be considered as an advocate of technology by my colleagues.	4.04 (0.86)		4.17 (0.92)		+0.13 (−0.23 to 0.48)		.47	
**FC^i^**									
	FC1: All the necessary resources to use the platform will be easily available.	3.33 (1.13)		4.00 (1.02)		+0.67 (0.22 to 1.11)		.007	
	FC2: The platform will not be expensive for our clinic to purchase.	3.75 (1.03)		4.21 (0.83)		+0.46 (0.01 to 0.91)		.046	
	FC3: The platform can be used with our existing infrastructure.	3.88 (0.95)		4.13 (0.85)		+0.25 (−0.24 to 0.74)		.21	
	FC4: Charging the smartphone will not be a challenge, even during outreach missions.	3.75 (0.99)		4.29 (0.75)		+0.54 (0.11 to 0.97)		.02	
	FC5^c,d^: The platform cannot be used in the current setting.	—		3.63 (1.21)		—		—	
**BI^j^**									
	BI1: If made readily available, I intend to use the platform in the near future.	4.08 (0.50)		4.67 (0.48)		+0.58 (0.34 to 0.83)		<.001	
	BI2: When available, I would like to take the platform along with other kits for outreach missions.	3.96 (0.62)		4.50 (0.59)		+0.54 (0.21 to 0.87)		.005	
	BI3: I am looking forward to using this platform when it is available.	4.00 (0.42)		4.67 (0.48)		+0.67 (0.46 to 0.87)		<.001	

^a^Wilcoxon signed-rank test (nonparametric).

^b^PE: performance expectancy.

^c^Inverted items.

^d^Extra item included only in the postuse questionnaire.

^e^Item included in preuse only or postuse only and data not available.

^f^EE: effort expectancy.

^g^FDT: frequency doubling technology.

^h^SI: social influence.

^i^FC: facilitating condition.

^j^BI: behavioral intention.

All UTAUT items captured responses via a 5-point Likert scale with responses that ranged from *strongly disagree* (1) to *strongly agree* (5; Multimedia Appendix 1). Upon completion of the preuse questionnaire, OPs could proceed to the next step of using GES. At the beginning of the visual field test with the GES, a brief demonstration of 5 minutes was given to each OP on how to use the GES. Upon request, support was provided only for three cases because of exceptional situations. At the end of the visual field test with GES, OPs completed a posttest questionnaire, followed by an interview to assess their attitudes (Multimedia Appendix 2).

The posttest questionnaire UTAUT items were slightly rephrased to reflect experience after use. Three additional items were included in the postquestionnaire relating to the actual experience of using GES: ease of administering the test to a patient, adequacy of the test result for making referral decisions, and whether GES could be used in the current setting (Table 2, items EE7, PE5, and FC5).

### Data Analysis

#### Quantitative Data

Of the 22 items in the preuse questionnaire, 6 items had a 4% (participants: 1/24) missing response rate, 4 items had an 8% (2/24) missing response rate, and 1 item had a 12% (3/24) missing response rate. Of the 25 items in the postuse questionnaire, 2 items had 4% (1/24 of participants) of missing responses and 3 items had 8% (2/24 of participants) of missing responses. All missing responses were replaced using the median imputation method [37]. Next, the data were checked for normality, skewness, and kurtosis [37]. Only one item (EE2) in the postuse questionnaire had a kurtosis issue with the absolute value of the kurtosis being slightly greater than three times the SE. Next, the internal consistency of the constructs was determined using Cronbach *α*. Unfortunately, for several constructs, these did not show sufficient internal reliability (Cronbach *α*<.7) [38]. Therefore, a decision was made to report results at the item level rather than at the construct level.

To assess whether attitudes changed significantly after use, the Wilcoxon signed-rank test was used to determine the significance of change in the scores (two-tailed, 95% CI; *α*=.05), before and after use of GES. As this study was exploratory in nature, there was no correction for family-wise error inflation. Instead, we have provided CIs that allow readers to obtain a direct understanding of size and effect. SPSS and Microsoft Excel were used for data analysis, and the Seaborn Python data visualization library [39] was used to illustrate data distribution with violin plots.

#### Qualitative Data

Qualitative data were obtained through interviews. Initial interview questions pertaining to the four UTAUT constructs were prepared. Additional questions were included for every respondent based on our observations during the screening test. Respondents were also invited to provide general feedback on GES at the end of the interview. The interviews were conducted immediately after the use of the GES to ensure that respondents had fresh memory of the use experience. All interviews were audio-recorded and transcribed verbatim. Interview data were categorized according to the major UTAUT constructs. Additional comments from OPs that did not fit any of the categories were handled separately, as comprehensive feedback on GES.

## Results

Here, we present the quantitative results for each of the five categories, along with the qualitative findings.

### PE Results

**Figure 5:**
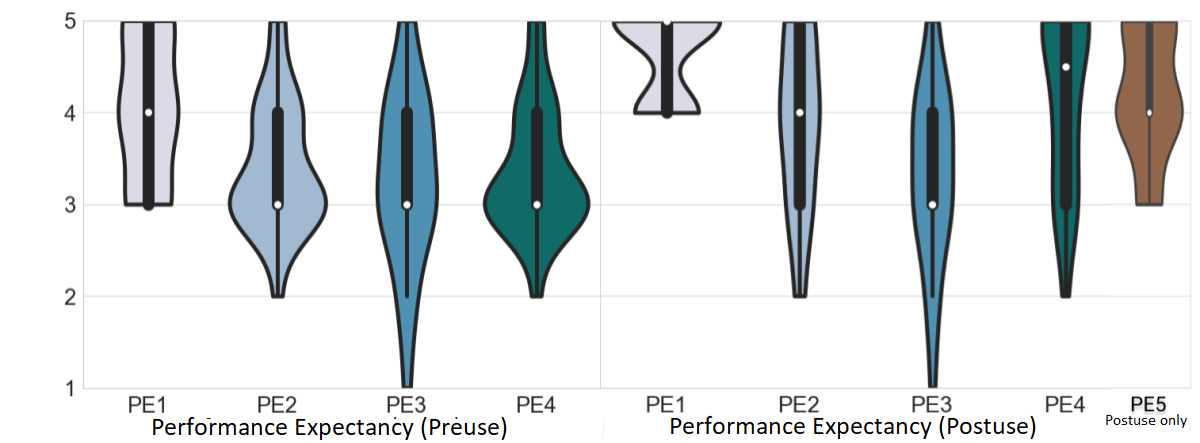
Performance expectancy scores for preuse are shown on the left, and those for postuse are shown on the right. The white dot represents the median value, the black bar represents the IQR, and the thin black bar denotes the 95% CI. PE: performance expectancy.

All preuse PE items scored above 3 (neutral), with PE1 (“this platform will be useful during outreach screening”) scoring the highest, with a mean of 4.04 (SD 0.81), and PE3 (“I do not expect that this platform can be used for a reliable eye exam”) scoring the lowest, with a mean of 3.29 (SD 0.95; Figure 5). After use, all PE items scored above 3; again, PE1 scored the highest value, with a mean of 4.63 (SD 0.49), and PE3 scored the lowest, with a mean of 3.33 (SD 0.96; Figure 5; postuse). Moreover, all PE items increased their score in postuse; items PE1 and PE2 reached significance, whereas PE3 and PE4 did not reach significance (Table 2).

From the interviews, we learned that although OPs supported the portability of GES for screening during field campaigns, such as the outreach mission, they also expressed their concern about its accuracy:

I think it will be useful because you can’t carry the FDT [Frequency Doubling Technology] to the outreach mission, but this one is easily portable. You can refer patients you suspect during screening. But I have no idea about its sensitivity and specificity.

P15, ophthalmic resident

If the result is valid about its sensitivity and specificity, I think it is informative enough. But it would be good if it includes something like a percentage of the defect, which will allow us to have a kind of cut point where we can say it is fine. If it is below that and more critical if it is above that level. Another important issue is...in rural areas, you may not have the other tools like an ophthalmoscope to examine the optic nerve. So, any macular disease or scar could cause visual field defect. So, you can’t immediately conclude that the defect is due to glaucoma.

P7, optometrist

### EE Results

**Figure 6:**
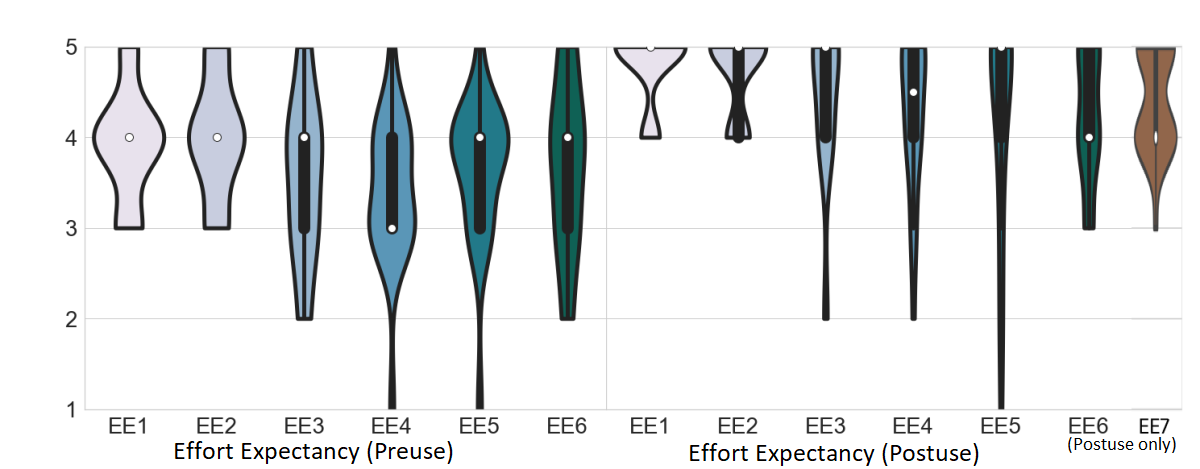
Effort expectancy scores for preuse are shown on the left, and those for postuse are shown on the right. The white dot represents the median value, the black bar represents the IQR, and the thin black bar denotes the 95% CI. EE: effort expectancy.

All preuse EE items scored above 3 (neutral), with EE2 (“I think learning how to use the platform will be easy”) scoring the highest, with a mean of 4.00 (SD 0.72), and EE3 (“It will be easy to have patients wear the VR headset”) the lowest, with a mean of 3.63 (SD 0.92; Figure 6; preuse). Postuse, all EE items again scored above 3, with EE1 (“I think the platform will be easy for me to administer visual field tests”) scoring the highest value, with a mean of 4.79 (SD 0.41), and EE5 scoring the lowest (:The platform will be easier for patients from rural areas compared with the FDT”), with a mean of 4.25 (SD 1.19; Figure 6 postuse). Moreover, all EE items increased their scores after use and reached significance (Table 2).

There were no specific difficulties expressed during the interviews related to administering the test to the patients:

This is easy for the patient. The joystick is good for the patient because it is possible to press any of the buttons. It won’t be a problem even if the patient presses another button by mistake. In addition, its cost very little.... For instance, you can’t carry the FDT to rural areas but this one is portable.

P6, ophthalmic resident

For me, it is almost similar. Your tool has a central target, the FDT also has a similar target. So, I don’t see much difference. But in terms of professional effort, I think this platform may be simpler [than the FDT] when we get used to it in the future.

P1, ophthalmic resident

What surprises me is when I read books they mention the FDT as a portable perimeter compared to Humphrey. But this is really portable.

P17, ophthalmic resident

### SI Results

**Figure 7:**
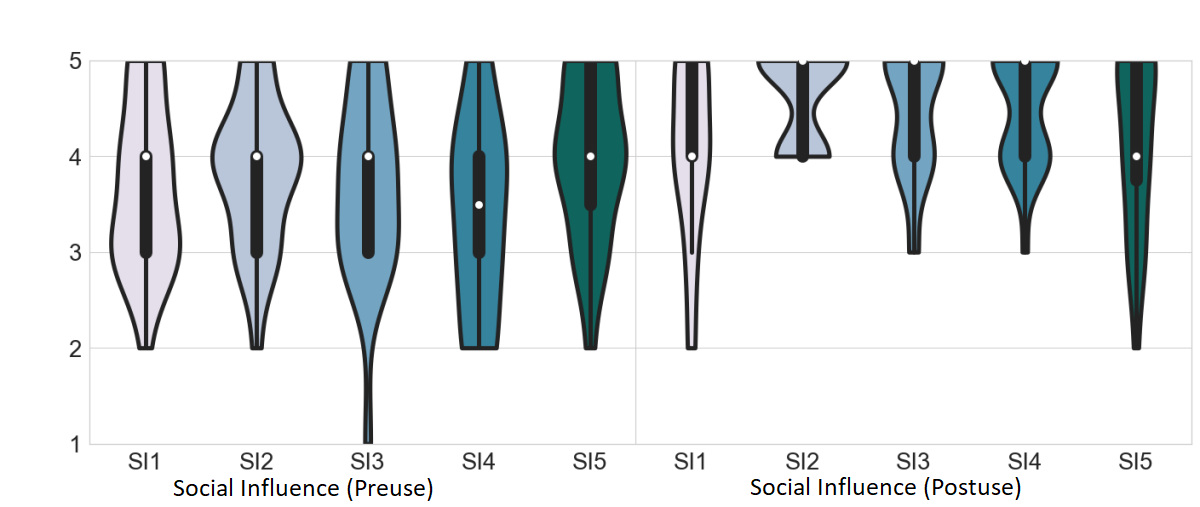
Social influence scores for preuse are shown on the left, and those for postuse are shown on the right. The white dot represents the median value, the black bar represents the IQR, and the thin black bar denotes the 95% CI. SI: social influence.

All preuse SI items scored above 3 (neutral), with SI5 (“If I use the platform, I will be considered as an advocate of technology by my colleagues”) scoring the highest, with a mean of 4.04 (SD 0.86), and SI4 (“My patients will be open to the use of this platform”) scoring the lowest, with a mean of 3.46 (SD 0.98; Figure 7; preuse). After use, all SI items scored above 3, with SI2 (“In general, I think the platform will be seen as useful by the ophthalmology department”) scoring the highest value, with a mean of 4.63 (SD 0.49), and SI1 (“I think the senior ophthalmologist staff will recommend the platform for glaucoma screening”) scoring the lowest, with a mean of 4.08 (SD 0.93; Figure 7 postuse). Moreover, all SI items increased their postuse scores. All items reached significance except SI1 and SI5, which did not reach significance (Table 2).

All OPs were confident about GES acceptance by colleagues and patients. Some OPs were doubtful about the support from hospital management.

We don’t have any worry about that [patients’ acceptance]. They will even feel like being diagnosed or examined more in detail. For instance, patients feel more treated when we examine them with slit lamp instead of torch light, even though the finding could be the same.

P21, optometrist

I am not sure about the hospital management, but our department will for sure support it.

P2, ophthalmic nurse

### FC Results

**Figure 8:**
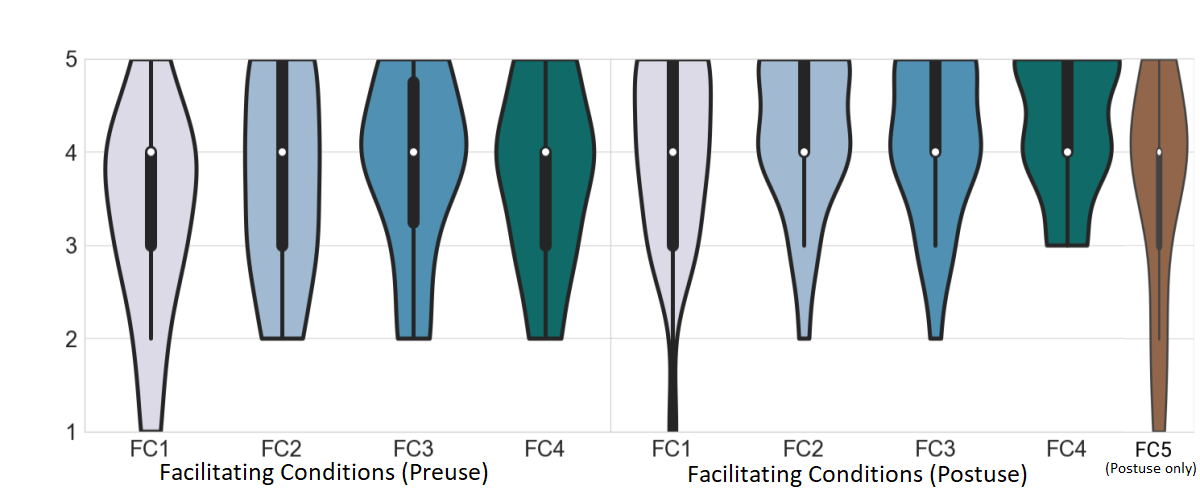
Facilitating condition scores for preuse are shown on the left, and those for postuse are shown on the right. The white dot represents the median value, the black bar represents the IQR, and the thin black bar denotes the 95% CI. FC: facilitating condition.

All preuse FC items scored above 3 (neutral), with FC3 (“The platform can be used with our existing infrastructure”) scoring the highest, with a mean of 3.88 (SD 0.95), and FC1 (“All the necessary resources to use the platform will be easily available”) scoring the lowest, with a mean of 3.33 (SD 1.13; Figure 8; preuse). After use, all FC items scored above 3, with FC1 scoring the highest value, with a mean of 4.29 (SD 0.75), and FC1 scoring the lowest, with a mean of 4.00 (SD 1.02; Figure 8 postuse). Moreover, all FC items increased their postuse scores. All items reached significance except FC3, which did not reach significance (Table 2).

There were no major concerns raised during the interviews relating to this category, with OPs showing optimism about its affordability and the possibility of charging the smartphone even in the worst conditions of having no access to electricity by using solar chargers:

I don’t think there will be a challenge with the battery. The worst case we can use solar chargers which are very common nowadays.

P8, ophthalmic resident

I think this can be even affordable by the ophthalmologists to buy one for their own let alone the institutions. They can also use it in their private clinic.

P13, cataract surgeon

### BI Results

**Figure 9:**
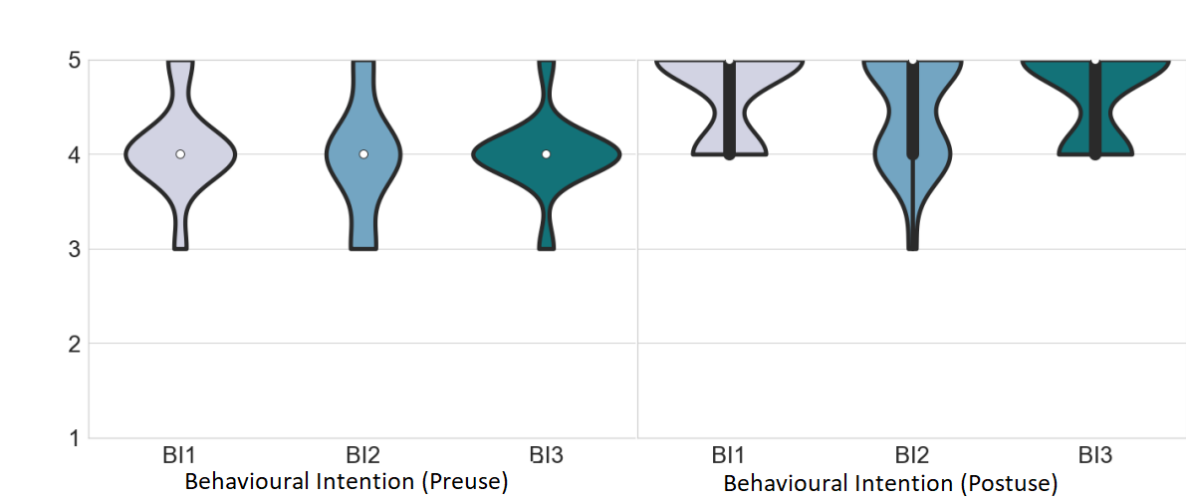
Behavioral intention scores for preuse are shown on the left, and those for postuse are shown on the right. The white dot represents the median value, the black bar represents the IQR, and the thin black bar denotes the 95% CI. BI: behavioral intention.

All preuse BI items scored above 3 (neutral), with BI1 scoring the highest, with a mean of 4.08 (SD 0.50), and BI2 scoring the lowest, with a mean of 3.96 (SD 0.62; Figure 9; preuse). After use, all BI items scored above 3, with BI1 and BI3 scoring the highest, with a mean of 4.67 (SD 0.48), and BI2 scoring the lowest, with a mean of 4.5 (SD 0.59; Figure 9 postuse). Moreover, all BI items increased their postuse scores. All items reached significance (Table 2).

Many OPs expressed their intention to use the system in the future, mentioning the dire need for a portable visual field screening tool:

I think we will use it as it is easy and very quick. The major reason we don’t use FDT for many of the patients is because of the duration we spend on a patient. If patients can easily understand it, we will for sure use it.

P19, ophthalmic resident

I am ready to use it because glaucoma is a challenge, especially during outreach missions. We refer patients by examining them only with an ophthalmoscope.

P2, ophthalmic nurse

### Summary of Qualitative Findings

#### Feasibility of Low-cost VR and Game Controllers

During this study, there were no significant difficulties in learning to use the GES. OPs were able to administer the test to a patient following a brief demonstration. Although for most patients, it was their first experience of wearing a VR headset and using a joystick, all of them were able to understand the test procedure and performed the test successfully in the first attempt. We observed that even older patients from rural areas, with limited familiarity with technology, were able to perform the test with GES based on the explanation given by OPs.

Moreover, ophthalmic nurses, optometrists, and IECW nurses at the primary hospitals saw an additional advantage in using GES, compared with the OPs at Jimma University Medical Center tertiary hospital. With almost no attention given to eye care at the primary level in the Ethiopian health system [36], they usually suffer from a lack of resources, including testing equipment. They usually rely on simple and basic tests with torch lights and simple magnifying lenses. They believed that when tested with a kit like GES, patients would be more satisfied because of a *feeling* of being better assessed with an instrument:

Patients are happier when examined with instruments. Even using the simple loop lens makes a lot of difference for patients. So, if we examine them with this tool, they might feel like they have been tested not only for glaucoma but for all kinds of eye problems

P24, IECW nurse

They will even feel like being diagnosed or examined more in detail. For instance, patients feel more treated when we examine them with slit lamp instead of torch light even though the finding could be the same. Patients will consider this a more advanced instrument which will give them a psychological satisfaction of feeling well treated.

P21, optometrist

#### Useful for Screening but Not for Diagnosis

In this study, the 2 IECW nurses at Seka Primary Hospital, who had limited ophthalmic training, had no difficulties in using the GES. However, when it came to interpreting the results of the visual field screening test, they expressed reservations. They believed that the actual identification of glaucomatous visual field defects required further training. A similar concern was also raised by ophthalmic residents at Jimma University Medical Center tertiary hospital during the postuse interviews. Although they believed that GES could improve the capacity of nurses at primary level health centers in detecting glaucoma cases, they recommended that its deployment be accompanied by a training on how to interpret results to make referral decisions. They also indicated the risk of non-OPs misusing it as a diagnostic tool to prescribe glaucoma medication to patients. They insisted that GES use should be limited to screening and not diagnosis, whereas also articulating that it should fulfill the criteria for clinical accuracy:

The procedure to use it was very simple for us to perform. But we don’t have any clue as to how to make conclusions based on the results. We need additional knowledge for that.

P24 and P25, IECW nurses

A concern I have is regarding the knowledge gap regarding visual field in general hospitals and health centers. Health professionals (non-ophthalmic) need training before using this platform. They might misinterpret the results and use it as a shortcut to prescribe medications to the patient. Remember this is a screening tool, not a diagnosis tool. So, the tool might be abused.

P8, ophthalmic resident

#### Precising the Desired Accuracy of the Tool

In our sample, there were differences among OPs regarding the acceptable level of accuracy for a screening test. Only 3 OPs expected a VFT result similar to that of SAP and frequency doubling technology equipment, whereas others considered a lesser accuracy (as currently obtained by GES [40] to be adequate and even more suited for screening purposes):

If the result is valid about its sensitivity and specificity, I think it is informative enough.

P7; optometrist

The information on the result is enough as to me. Remember this will most probably be used by non-ophthalmic professionals. Adding other details may not add any value since they don’t have any knowledge. I think the basic information are included.

P19, ophthalmic resident

With the FDT we have get details like the standard deviation, pattern standard deviation and others which are important for glaucoma diagnosis. But for this one we don’t know the details.

P15, ophthalmic resident

#### Integrating With Patient Records

Finally, the last topic discussed was the integration of the data given by GES with the current traill of patient data. There were also differences among OPs on how to integrate GES test results with the existing paper-based patient record management at the hospitals. Four OPs suggested the possibility of integration with a future digital health record system, whereas others considered it sufficient to obtain results on the smartphone screen, similar to their experience during this study. Overall, the interviews suggested that this part of health care has yet to mature and that challenges in current infrastructure in low-income countries may still warrant a tangible output:

There is a new technology coming to get rid of the paper documents and automate the patient record keeping. If that system is in place the app can easily be integrated with the system. But with the current setup there should be a paper print out to be enclosed in the patient’s folder.

P4, optometrist

Since it is a screening tool, not a diagnosis tool, as to me there is no need for a printout because I will still have the result on the mobile even if the patient folder is lost.

P3, ophthalmic resident

## Discussion

### Principal Findings

Glaucoma, which is also referred to as *the silent thief of sight* [41], is a major cause of irreversible blindness worldwide [42], particularly in Africa where it accounts for 15% of all blindness. As glaucoma screening and diagnosis depend on expensive, stationary equipment, timely eye examination is almost impossible for people living in rural areas of ow-income countries like Ethiopia. This study assessed the acceptance of a low-cost and portable glaucoma screening solution through UTAUT-based surveys, pre and postuse in 4 different hospitals in Southwest Ethiopia. Positive results were obtained preuse, suggesting acceptance of mHealth solutions for glaucoma screening, using a low-cost headset through a smartphone and a game controller. Moreover, almost all scores improved significantly after use, particularly after using GES. OPs perceive GES as a handy and easy-to-use kit in their fight against glaucoma (EE). They believed that GES would enhance the screening of glaucoma (PE), especially during outreach field missions to rural areas because of its portability and short test duration. They also considered GES to be usable within the existing health care system (FCs) and believed that its use would be favored by their colleagues and management (SI). The positive scores of the different drivers were reinforced by the high scores of their BI to use GES in their daily practice. Complementing the quantitative results, the observations and interviews confirmed the enthusiasm for using GES in the future.

Perceived ease of use (EE) has been identified as one of the major factors influencing mHealth adoption by health professionals [19,20,36]. One of the major challenges Ethiopian OPs face while administering VFT is the extra effort required to explain the test procedure to patients from rural areas with little familiarity with technology, to the extent of not even having experience in using a TV remote control [36]. Both quantitative and qualitative results indicate that GES is easy to use, even for patients from rural areas. This reinforces the findings of Lodhia et al [24] and Ludwig [25] that patients in rural areas are able to overcome the barriers to using technologies for ophthalmic services. Hence, these findings suggest that cultural beliefs or lack of technological ineptitude, often mentioned as barriers to adoption [17,18], will not hamper acceptance of GES. In summary, this supports the feasibility of using a mobile device in combination with a classic controller and a low-cost VR headset.

Given the dire shortage of OPs in rural areas of low-income countries [5], mHealth solutions for eye care, such as GES, aim to allow for examinations to be performed by less trained or educated health care workers, such as nonophthalmic nurses. The major concern of highly trained OPs at the tertiary hospitals was that GES might be misused for diagnosis by less trained nurses at the primary level, in addition to screening. This finding contrasts with the study by Chang et al [43], who identified concerns of confidentiality of data and worry over job security. Our study suggests that OPs’ concerns are mainly focused on inappropriate use of the tool, not job erosion. Hence, any mHealth solution for eye health care should carefully strike a balance between empowering primary care level workers who have less specialized training and enabling abuse, overstepping from screening to unwarranted diagnosis.

Similar to findings by Lodhia et al [24], in which health professionals expressed the need to determine the clinical accuracy of polyetheretherketone before its use as a screening tool, in th study, OPs recommended further research on sensitivity and specificity. This highlights that positive scores on the PE were given *conditionally*, under the assumption that clinical accuracy would be satisfactory.

### Limitations and Future Work

Although this study provides interesting insights into the factors that influence OP acceptance of a mhealth solution for eye care, it has limitations. First, given the dire shortage of OPs in the southwest region of Ethiopia, the sample size was small. Second, OP perceptions after use were measured after a single use of GES. Hence, we were not able to measure their perspectives based on long-term, continued use. Therefore, possible additional challenges not identified in this study may still arise during long-term use. Last, as we adapted items from the original UTAUT questionnaires and added our own items, internal consistency at the construct level was no longer achieved (Cronbach *α*<.7 for two constructs in preuse and four constructs in postuse). This necessitated quantitative analysis for individual items and may introduce an overlall less robust measurement. Several studies support the analysis of individual items instead of constructs in these situations. For example, Aljarallah S and Lock R [44] report on users’ perspective toward software sustainability and present much of their analysis at the item level despite reporting a Cronbach *α* of <.7 for some of the constructs, while Marchewka JT and Kostiwa K [45] reported much of their descriptive analysis from a UTAUT-driven questionnaire at an item level after presenting construct level reliability measures. Furthermore, Elo A-L et al [46] and Clason et al [47] demonstrated the validity of analyzing individual survey items (not summated). We hope to address this issue by providing descriptive results for all items.

### Conclusions

This paper presents a mixed method study on the acceptance and use of a low-cost and portable smartphone-based visual field screening tool for glaucoma (GES) at 4 hospitals in Southwest Ethiopia. We combined the survey data and interviews of 24 OPs. Both the quantitative and qualitative results suggest high levels of acceptance. Additionally, this study provided better insight into the factors influencing health professionals’ acceptance of mHealth interventions for eye care in l-income countries such as Ethiopia. We found that OPs perceived GES as easy to use, enabling the conduct of glaucoma screening tests, especially during outreach to rural areas. Even older patients from rural areas, with limited familiarity with technology, were able to perform the test with GES and valued a *technical* assessment. Nevertheless, as frequently indicated by the OPs, a further assessment of its sensitivity and specificity is needed, as positive evaluations are contingent on the assumption of adequate accuracy. Moreover, this tool is suitable for screening and not for diagnosis. Accounting for these caveats, GES has the potential to reduce the burden of irreversible blindness caused by glaucoma.

## Appendix

Multimedia Appendix 1 Questionnaire.

Multimedia Appendix 2 Semistructured interview guide.

## Abbreviations

BI: behavioral intention
EE: effort expectancy
FC: facilitating condition
GES: Glaucoma Easy Screener
IECW: integrated eye care worker
mHealth: mobile health
OP: ophthalmic professional
PE: performance expectancy
SAP: standard automated perimetry
SI: social influence
UTAUT: Unified Theory of Acceptance and Use of Technology
VFT: visual field testing
VR: virtual reality
