# Supplementary material for: Acceptance of a Smartphone-Based Visual Field Screening Platform for Glaucoma: Pre-Post Study
Source: JMIR Form Res. 2021 Sep 17;5(9):e26602. doi: 10.2196/26602 (PMC8486992; doi:10.2196/26602)
Supplement: Multimedia Appendix 1 [file formative_v5i9e26602_app1.pdf]

## Multimedia Appendix I: Questionnaire

| Usability of the platform(put a tick(✓) for the answer of your choice)-PRIOR | | | | | | | |
|---|---|---|---|---|---|---|---|
| | | Strongly Disagree | Disagree | Neutral | Agree | Strongly Agree |
| PE1 | This platform will be useful during outreach screening. | | | | | |
| EE1 | I think the platform will be easy for me to administer visual field tests. | | | | | |
| SI1 | I think the senior ophthalmologist staff will recommend the platform for glaucoma screening. | | | | | |
| FC1 | All the necessary resources to use the platform will be easily available. | | | | | |
| BI1 | If made readily available, I intend to use the platform in the near future. | | | | | |
| PE2 | The platform will enable me to make more accurate diagnosis. | | | | | |
| EE2 | I think learning how to use the platform will be easy | | | | | |
| SI2 | In general, I think the platform will be seen as useful by the ophthalmology department. | | | | | |
| FC2 | The platform will not be expensive for our clinic to purchase. | | | | | |
| BI2 | When available, I would like to take the platform along with other kits for outreach mission. | | | | | |
| FC3 | The platform can be used with our existing infrastructure | | | | | |
| SI3 | My colleagues will support my use of this platform | | | | | |
| SI4 | My patients will be open to the use of this platform | | | | | |
| EE3 | It will be easy to have patients wear the VR headset | | | | | |
| PE3 | I do not expect that this platform can be used for a reliable eye exam. | | | | | |
| EE4 | Compared to the FDT, administering visual field testing with this platform will be easier | | | | | |
| BI3 | I am looking forward to using this platform when it is available. | | | | | |
| FC4 | Charging the smartphone will not be challenge, even during outreach missions. | | | | | |
| SI5 | If I use the platform, I will be considered as an advocate of technology by my colleagues. | | | | | |
| EE5 | The platform will be easier for patients from rural areas compared to the FDT | | | | | |
| PE4 | More glaucoma cases can be detected by the platform. | | | | | |
| EE6 | Patients can easily use a joystick for response. | | | | | |

| **Usability of the platform(put a tick(✓) for the answer of your choice)-POST** | | | | | | |
|---|---|---|---|---|---|---|
| | | Strongly Disagree | Disagree | Neutral | Agree | Strongly Agree |
| EE1 | The platform is easy for me to administer visual field tests. | | | | | |
| SI1 | In general, I think the platform will be seen as useful by the ophthalmology department | | | | | |
| FC1 | All the necessary resources to use the platform are easily available. | | | | | |
| BI1 | If made readily available, I will intend to use the platform in the near future. | | | | | |
| PE1 | The platform will enable me to make more accurate diagnosis. | | | | | |
| EE2 | It was easy to make patients wear the VR headset. | | | | | |
| SI2 | My colleagues will support my use of this platform. | | | | | |
| FC2 | Charging the smartphone will not be a challenge, even during outreach missions. | | | | | |
| BI2 | I am looking forward to using this platform when it is available. | | | | | |
| EE3 | Learning how to use the platform was easy for me. | | | | | |
| PE2 | I do not expect that this platform can be used for a reliable eye exam. | | | | | |
| EE4 | Compared to the FDT, administering visual field with this platform is easier. | | | | | |
| SI3 | If I use the platform, I will be considered as an advocate of technology by my colleagues. | | | | | |
| FC3 | The platform cannot be used in the current setting. | | | | | |
| BI3 | I would like take the platform along with other kits for outreach mission, when available. | | | | | |
| EE5 | The platform is easier to use with patients from rural areas compared to the FDT. | | | | | |
| PE3 | More glaucoma cases can be detected by the platform. | | | | | |
| EE6 | Patients could easily use the joystick to give their response. | | | | | |
| SI4 | The senior ophthalmologist staff is likely to recommend the platform for glaucoma screening. | | | | | |
| FC4 | The platform is not expensive for our clinic to purchase. | | | | | |
| EE7 | My interaction with the platform was clear and understandable. | | | | | |
| PE4 | The results presented are informative enough to make referral decisions. | | | | | |
| FC5 | The platform can be used with our existing infrastructure. | | | | | |
| PE5 | This platform will be useful during outreach screening. | | | | | |
| SI5 | My patients will be open to the use of the platform. | | | | | |
