# Supplementary material for: Acceptance of a Smartphone-Based Visual Field Screening Platform for Glaucoma: Pre-Post Study
Source: JMIR Form Res. 2021 Sep 17;5(9):e26602. doi: 10.2196/26602 (PMC8486992; doi:10.2196/26602)
Supplement: Multimedia Appendix 2 [file formative_v5i9e26602_app2.pdf]

Multimedia Appendix II: Semi-Structured Interview Guide

- Performance Expectancy
  - Do you think this is a useful platform? Why? Why not?
  - How do you compare this product with FDT?
  - Is the test duration acceptable?
  - Is there enough time to use the platform for screening during normal consultation hours or outreach mission.
  - Are the data presented on the test result appropriate? Anything to omit or include?
  - Do you think it is suitable for glaucoma patients?
  - What concerns do you have if this product is to be made ready for clinical use?
  - How do you suggest the results to be included in the dossier of the patient?
  - How does the demo app help in explaining the test to the patient?
  - Any feature missing to make it more useful?
- Effort Expectancy
  - In general, did you find this easy to use? Why? Why not?
  - Is it more easier to explain compared to the FDT test?
  - Was it easy to mount the headset to the patient?
  - Wat it easy to interpret the results?
- Facilitating Conditions
  - Do you think the current infrastructure is good enough to use this technology? Why? Why not?
  - Do you think this product can be used in a busy environment with many patients waiting?
  - Are there other environmental constraints that may complicate the use?
  - How would you charge the battery? Where would you store it?
- Social Influence
  - How will colleagues perceive this platform?
  - Do you think management will support it? Why? Why not?
  - Will patients be willing to have them screened by this system?
- Behavioral Intention
  - Would you like to you use it in the future? Why? Why not?
- General
  - Do you have any additional comments?
